# Microbiological quality of mink feed raw materials and feed production area

**DOI:** 10.1186/s13028-019-0489-6

**Published:** 2019-11-21

**Authors:** Ulrike Lyhs, Henrik Frandsen, Birgitte Andersen, Bettina Nonnemann, Charlotte Hjulsager, Karl Pedersen, Mariann Chriél

**Affiliations:** 1 National Veterinary Institute, Technical University of Denmark, Kemitorvet, Building 202, 2800 Kgs. Lyngby, Denmark; 2 National Food Institute, Technical University of Denmark, Kemitorvet, Building 202, 2800 Kgs. Lyngby, Denmark; 3 Department of Biotechnology and Biomedicine, Technical University of Denmark, Søltofts Plads, Building 221, 2800 Kgs. Lyngby, Denmark; 4 Department of Animal Health and Antimicrobial Strategies, National Veterinary Institute, Ulls väg 2B, 751 89 Uppsala, Sweden; 5 Present Address: Institute for Molecular Medicine Finland (FIMM), University of Helsinki, P.O.Box 20, Tukholmankatu 8, 00014 Helsinki, Finland; 6 Present Address: Statens Serum Institut, Artillerivej 5, 2300 Copenhagen S, Denmark; 7 Present Address: Environmental Protection Agency, Tolderlundvej 5, 5000 Odense C, Denmark

**Keywords:** *Aspergillus*, *Clostridium*, Hygiene, Influenza A virus, Mink, Mycotoxins

## Abstract

**Background:**

The quality of mink feed and raw ingredients affect health and growth. The objectives of this study were to examine the microbiological quality of ready-to-eat mink feed and its raw ingredients, screen the plant part of the feed for mycotoxins, and determine the hygiene of the production environment in the feed processing facilities. The results of the study are important for identification of critical steps in the feed production and for formulation of recommendations for improvements of production processes to obtain better quality feed. Feed and swab samples were taken at three Danish mink feed producers October 2016 and May 2017, respectively. Viable counts, detection of methicillin-resistant *Staphylococcus aureus* (MRSA), influenza virus and filamentous fungi were performed together with qualitative chemical analyses for bioactive fungal metabolites and mycotoxins. Swab samples were analyzed for total viable counts.

**Results:**

Viable counts varied between 7.2 × 10^2^ and 9.3 × 10^7^ cfu/g in raw ingredients and between 10^7^ and 10^9^ cfu/cm^2^ on different surfaces at the feed production facilities. A pork meat product, pork haemoglobin, pork liver and a poultry mix was found positive for MRSA, while monophasic *Salmonella* [4,5,12:i:-] was detected in a pork meat product. Neither MRSA nor *Salmonella* was detected in any ready-to-eat feed. Influenza A virus was not detected in any sample. Filamentous fungi were detected in all analysed samples of ready-to-eat feed while dihydro-demethyl-sterigmatocystin was found in almost 50% of all ready-to-eat feed samples and in 80% of the sugar beet pulp. Fumonisins and other *Fusarium* toxins were found especially in corn gluten meal and extruded barley and wheat.

**Conclusions:**

Mink feed contained a cocktail of mycotoxins and bacteria, which may not per se cause clinical disease, but may affect organ function and animal performance and well-being.

## Background

Denmark is the world’s largest producer of mink skins. The American mink (*Neovison vison*) is used for livestock production with 1248 commercial mink farms registered in 2018, which produced approximately 17.2 million mink skins (https://statistikbanken.dk). Feed factories supply the mink farms with pumpable wet feed consisting of a mix of animal by-products and slaughter offal (e.g. by-product from the fishing and meat industries) and plant origin (e.g. corn gluten meal, soybean oil and extruded cereals).

Raw ingredients of animal origin, mainly fish and poultry products and their by-products, are susceptible to bacterial contamination and spoilage. Likewise, ingredients of plant origin are susceptible to fungal spoilage and synthesis of mycotoxins. Spoilage microorganisms will always impair the quality, digestibility and the nutritional value of the feed and consequently, reduce not only the productivity of the animals, but also their health, behaviour and welfare. Limited work has been carried out to investigate the microbiological quality of mink feed. When analysing bacterial counts including enterococci, coliforms, haemolytic and sulphite-reducing bacteria in different raw materials, Juokslahti [1] reported high bacterial counts in fish by-product and blood meal with slaughterhouse by-product and unpreserved slaughter blood having the poorest quality.

The ready-to-eat feed may contain pathogenic or toxigenic bacteria, fungi and viruses originating from contaminated raw materials, which may cause diseases in the mink. Dietz et al. [2] described *Salmonella* Dublin-associated abortion in Danish mink and fox farms concluding that contaminated feed was responsible for the outbreaks. Other studies described uterine infections of mink caused by *Clostridium limosum* with suspicion of feed-borne transmission [3]. Hammer et al. [4] reported a spontaneous outbreak of abortions and mortality in Danish mink farms probably due to *C. limosum* found in raw mink feed samples. Hansen et al. [5] found livestock-associated methicillin-resistant *Staphylococcus aureus* (LA-MRSA) in 20 out of 58 (34%) of clinical mink samples and in 20/108 (19%) of feed samples. Studies have shown mink to be sensitive to a number of mycotoxins, including aflatoxin B1, deoxynivalenol, zearalenone, fumonisins, moniliformin and ergot alkaloids [6]. Bursian et al. [6] also showed that mink lost weight and developed kidney damage when fed ochratoxin contaminated feed. Aflatoxin-containing feed can cause poor skin quality, weight loss and kidney and liver damage [7], while deoxynivalenol (DON, vomitoxin) and other Type B trichothecenes have been demonstrated to cause emesis in the mink [8].

Analyses of medication and feed quality showed that the consumption of antibiotics increased when the numbers of faecal cocci were high [9, 10] although a direct association needs to be further verified. Poor feed quality may therefore indirectly lead to increased antimicrobial use. An earlier study of gastrointestinal disorders in mink found that differences between the feed producers as a risk factor accounted for 80% of the variation in occurrence of gastrointestinal disorders [11]. Antibiotics are often used for treating unspecific diarrhoea or clinical pneumonia, and to a lesser extent in sporadic outbreaks of contagious diseases, such as *Pseudomonas aeruginosa,* and secondary infections after outbreak of mink influenza or canine distemper. However, resistance in the bacterial population is amplified by selection pressure from antibiotics use, which may result in reduced or—in worst case—no effect of the antibiotic treatment [12, 13].

Good quality of feed is essential for the animal performance, health and welfare. This applies to both nutritional value and microbiological quality. In the present study, we have focussed on the bacterial and mycological quality with the purposes to: (1) examine the microbiological quality of the raw ingredients (animal and plant origin) and the ready-to-eat mink feed; (2) screen the plant ingredients and ready-to-eat feed for mycotoxins and; (3) determine the hygiene level of the production environment in the feed processing facilities. The results may be important to identify any critical steps in the production of feed and may therefore be important to assist the feed factories in improving production processes to obtain better quality feed.

## Methods

### Collection and transport of the samples

Three Danish mink feed producers (coded A to C) were visited twice: when the feed production was at its maximum volume (late October 2016) and at its minimum (mid May 2017). From each producer, 20 samples from each raw ingredient of animal origin, five samples from each raw ingredient of plant origin and five samples from the final product (ready-to-eat feed) were collected on each visit. For each frozen sample type, the feed producer used a machine to drill a hole in the frozen pallets of feed ingredients. The cylinder of feed ingredient left on the drill was transferred to a sample container. Between each sample collection, the drill was rinsed with hot water (> 50 °C). For the all other sample types, samples were taken randomly from the feed ingredients and transferred to a sample container. They were placed in a cool box and transported immediately to the laboratory and subjected to bacterial analyses within 4 to 6 h. The number of sample types collected from all three mink feed producers during both sampling years and the number of analyses performed on them are indicated in Table 1.

**Table 1:** Type of sample, analyses and total number of samples taken from three mink feed producers during October 2016 and May 2017

Samples	Type of analyses	Number of samples
Raw ingredients of animal origin	Detection of bacteria	45
	Detection of influenza A virus	43
	Detection of fungal metabolites and mycotoxins	6
Raw ingredients of plant origin	Detection of fungal metabolites and mycotoxins	35
Ready-to-eat feed I	Detection of bacteria	6
	Detection of influenza A virus	6
	Detection of filamentous fungi	10
	Detection of fungal metabolites and mycotoxins	25
Ready-to-eat feed II	Detection of bacteria	6

In each sampling year, a total of 25 swab samples were taken from five different surfaces (10 × 10 cm) at all three feed producers. For transport to the laboratory, five swabs from the same surface were pooled to one subsample and placed into a sterile tube containing 10 mL of 0.9% NaCl (w/v) and 0.1% (w/v) peptone water and stored at 4 °C.

### Microbiological analyses

#### Detection of bacteria in samples of animal origin

Twenty-five gram of each raw feed ingredient of animal origin and of the ready-to-eat feed product (one pooled sample of five subsamples of 5 g each), respectively, were aseptically transferred to a Stomacher^®^ 400 bag (Seward BA6041, Worthing, UK) containing 225 mL of 0.9% NaCl (w/v) and 0.1% (w/v) peptone water. The bag was blended in a Stomacher 400 Lab Blender (Seward Medical, London, UK) for 3 min. Tenfold serial dilutions were used for microbiological analyses. Total viable counts (TVC) were performed on spread plates with Plate Count Agar (PCA) (Thermo Fisher Scientific, Roskilde, Denmark) according to method ISO 4833-2:2012 [14]. The number of *Enterobacteriaceae* was determined by pour plating in Violet Red Bile Glucose (VRBG) agar (Oxoid, Basingstoke, UK) according to the method of ISO 21528-2:2004 [15]. Counts of clostridia were done by pour plating method on Tryptose Sulfite Cycloserine (TSC) agar (Oxoid) by the method ISO 7937:2004 (E) [16]. *Escherichia coli* counts were determined on spread MacConkey agar plates (Thermo Fisher Scientific) using ISO 16649-2:2001 [17], except that we used MacConkey agar instead of 5-bromo-4-chloro-3-indolyl beta-d-glucuronide. The number of staphylococci were performed on spread plates with Baird-Parker agar (Thermo Fisher Scientific) according to the method of ISO 6888-1:1999 [18]. All PCA plates were counted after 3 days’ aerobic incubation at 30 °C. Typical *Enterobacteriaceae* colonies were counted after 18–24 h aerobic incubation at 37 °C. Both MacConkey and Baird-Parker plates were counted after 18–24 h and 2 days, respectively, aerobic incubation at 37 °C. TSC plates were counted after 18–24 h anaerobic incubation at 37 °C.

From each ready-to-eat feed product, one subsample (ready-to-eat feed I) was immediately analyzed for bacteria as described before (t = 0). In order to simulate handling of the final feed product before mink may eat the feed, a second subsample (ready-to-eat feed II) was stored in the sampling container with lid open in room temperature overnight and analyzed the next day (t = 24).

For comparison of viable counts between seasons and feed factories, viable counts were log-transformed and hereafter compared using two-sided t-test.

For detection of *Salmonella* spp. [19], 25 g each of raw feed ingredient of animal origin and ready-to-eat feed (t = 0 and t = 24), respectively, were aseptically transferred into a Stomacher^®^ 400 bag (Seward BA6041) containing 225 mL of buffered peptone water (BPW) (Thermo Fisher Scientific), then blended in a Stomacher 400 Lab Blender (Seward Medical) for 3 min and incubated at 37 °C for 16–20 h. Three drops (totaling 0.1 mL) of this BPW pre-enrichment culture were equally inoculated on Modified Semisolid Rappaport–Vassiliadis (MSRV) agar (Thermo Fisher Scientific) plates and incubated at 41.5 °C for 18–24 h. From MSRV, suspect growth was sub-cultivated on Brilliant Green Agar (BGA) (Thermo Fisher Scientific) and on Xylose Lysine Deoxycholate (XLD) Agar (Thermo Fisher Scientific). Similar to transferring BPW to MSRV plates, 1 mL of BPW was transferred into 9 mL Selenite Cystine (SC) broth (Thermo Fisher Scientific) and incubated for 18–24 h at 41.5 °C. From here, the BGA and XLD agar were inoculated. Suspect colonies from XLD and BGA were subcultured on Columbia agar plates containing 5% calf blood (SSI Diagnostica, Hillerød, Denmark) and verified by agglutination with polyvalent *Salmonella* serum (SSI Diagnostica).

#### Detection of methicillin-resistant *Staphylococcus aureus* (MRSA)

For detection of MRSA, raw feed ingredients of animal origin and of the ready-to-eat feed product were kept frozen until analysis. All samples were analysed by one-step enrichment, where 10 g were inoculated in 90 mL Mueller–Hinton Broth with 6.5% NaCl for 18–24 h at 37 °C without agitation. A loop-full (10 µL) enriched sample material was streaked on Brilliance MRSA2 agar (Oxoid) and incubated at 37 °C for 18–24 h. Presumptive MRSA colonies identified as denim blue colonies on MRSA2 agar were sub-cultured on agar plates (Oxoid) containing 5% calf blood for further verification. Isolates were identified as MRSA by PCR detection of the *mecA* and *nuc* genes [20].

#### Detection of filamentous fungi in ready-to-eat feed samples

For determination of filamentous fungi present in the feed, 1 g of thawed, ready-to-eat feed product was transferred directly to Dichloran 18% Glycerol agar (DG18 ([21])) and spread over the surface of the agar. Likewise, 1 g from each ready-to-eat product was plated onto vegetable juice agar (V8 ([21])). The plates were incubated in darkness at 20 °C and read after 5, 7 and 10 days. Representative fungi were isolated and inoculated onto different media for species identification according to Samson et al. [21]. In brief: *Aspergillus* cultures were 3-point inoculated onto CYA, MEA and DG18 and incubated in darkness at 25 °C and CYA at 37 °C. *Penicillium* cultures were 3-point inoculated onto CYA, MEA and YES and incubated in darkness at 25 °C and CYA at 30 °C. Zygomycetes were 1-point inoculated on MEA, OA and DG18 [21] and incubated in darkness at 25 °C and MEA at 30 °C.

#### Detection of bacteria in environmental samples

In the laboratory, 90 mL of 0.9% NaCl (w/v) and 0.1% (w/v) peptone water were added to each tube containing five pooled swabs and vortexed for 30 s. Tenfold serial dilutions were used for microbiological analyses. Total viable counts (TVC) were performed on spread plates with Plate Count Agar (PCA) (Thermo Fisher Scientific) as described above.

### Detection of influenza virus A

RNA was purified from all raw feed ingredients of animal origin (except blood meal and pork fat) and from ready-to-eat feed. Liquid samples were pre-treated by lysing 200 μL of sample in 400 μL of RLT Buffer (QIAGEN) with 1% β-mercaptoethanol while solid samples were homogenized by beating on a Tissue lyser II (QIAGEN) to prepare 10% homogenate in RLT Buffer (QIAGEN) with 1% β-mercaptoethanol. RNA was purified from 600 μL homogenate/lysate using RNeasy Mini Kit (QIAGEN) on a QIAcube (QIAGEN) purification robot with protocol animal tissues and cells, large samples, version 2. The RNA was tested for influenza A virus using real-time RT-PCR directed against the matrix gene [22]. In order to counteract possible PCR inhibition, the test was performed on both undiluted RNA and on RNA diluted tenfold in nuclease-free water.

### Chemical analyses

#### Detection of mycotoxins and other bioactive fungal metabolites

Acetonitrile, methanol, formic acid and 25% ammonium hydroxide (LC–MS grade) and ethyl acetate were obtained from Sigma-Aldrich, Schnelldorf, Germany. Evolute express ABN SPE columns were obtained from Biotage, Sweden. Water was purified on a Milli Q system (Millipore Corporation, USA). Aflatoxin B1, B2, G1, and G2, citrinin, cyclopiazonic acid, deoxynivalenol, enniatin A1 and B1, fumonisin B1 and B2, nivalenol, ochratoxin A, patulin, sterigmatocystin, T2 toxin and zearalenone were all obtained from Sigma-Aldrich.

#### Sample preparation

Sample material of 0.2 g (ready-to-eat feed or raw material) was transferred to a bead beater tube and added 1 mL 80% acetonitrile. After homogenization for 1 min the tube was cooled to 4 °C and centrifuged at 10,000×*g*. The supernatant was transferred to an Eppendorf tube and frozen at − 20 °C for 1 h followed by centrifugation at 10,000×*g* at 4 °C for 10 min. A SPE column (evolute express ABN 30 mg) was washed with 2 mL acetonitrile and dried and 0.3 mL of the supernatant was passed through the column and transferred to a HPLC vial.

#### Liquid chromatography quadrupole time-of-flight mass spectrometry

Liquid chromatography was performed on a Dionex Ultimate 3000 RS (Thermo Scientific) with a Poroshell SB C-18 column (100 mm length and 2.1 mm inner diam., 2.7 µm particle size) column held at 30 °C (Agilent Technologies, Walbron, Germany). The solvent system consisted of A: 2.5 mM ammonium hydroxide + 0.02% formic acid in water and B: methanol. Solvent programming were: 10% B at 0 min followed by a linear gradient to 45% B at 3 min and a linear gradient to 95% B at 14 min, isocratic 95% B from 14 to 16 min followed by reversal to initial conditions at 16.1 min and re-equilibration of the column to 20 min. The flow rate was 0.2 mL/min from 0 to 1 min followed by a linear gradient to 0.4 mL/min to 14 min, which was held until 16 min followed by reversal to initial conditions at 19 min.

The LC system was connected to a Bruker Daltonics, maXis qTOF mass spectrometer equipped with an electrospray ion source operated in positive ion mode (Bruker Daltonics, Bremen, Germany). The ion source settings were: nebulizer pressure 2 bars, drying gas flow 8 L/min, dry gas temperature 200 °C, and capillary voltage 4000 V. The scan range was from 30 to 1000 *m/z* with an acquisition rate of 2 Hz. Sodium formate dissolved in 50% 2-propanol was introduced in the ion source in a 0.2–0.4 min time segment and used for internal calibration of the data files. Hexakisperflouroethoxyphophazene was used as lock mass calibrant (Apollo Scientific, UK).

#### Fungal metabolite analyses

Data files were processed using Target Analysis (Bruker Daltonics). Based on information on the elemental composition of the analytes the software extracts ion chromatograms of the exact mass ± 3 mDa of aflatoxin B1, B2, G1, and G2, citrinin, cyclopiazonic acid, deoxynivalenol, enniatin A1 and B1 (ammonium adducts), fumonisin B1 and B2, nivalenol, ochratoxin A, patulin, sterigmatocystin, T2 toxin and zearalenone followed by integration of the chromatograms and reporting of the results.

#### pH-measurement

The pH was determined from the first homogenate made for microbiological analysis by a Sartorius PB 310 Digital-pH-meter, Type PHM 92, (Radiometer, Analytical A/S, Copenhagen, Denmark).

## Results

### Bacteriological analyses

The bacterial counts for each raw ingredient of animal origin and the ready-to-eat feed I and II from all three feed producers (A to C) in both sampling years 2016 and 2017 are shown in Additional files 1, 2, 3, 4, 5 and 6. Note that there are differences in sample categories between the years for the same producer.

In the raw ingredients from feed producer A in both sampling years 2016 and 2017, total viable count (TVC) between 1.8 × 10^4^ and 9.3 × 10^7^ colony-forming units/g (cfu/g) were found in most of the raw ingredients except in the fish silage (Additional files 1 and 4). In 2016, high *Enterobacteriaceae* counts were determined in raw ingredients from fish and pork products, but only low counts from poultry by-products (2.6 × 10^7^ cfu/g) in 2017. In 2017, samples from poultry by-products and fish cut showed *E. coli* counts of 4.7 × 10^7^ and 9.4 × 10^5^ cfu/g, respectively (Additional file 4). In the same year, counts of staphylococci up to 2.4 × 10^6^ cfu/g were detected in poultry by-products.

In 2016, TVC of all ingredients from feed producer B varied between 7.2 × 10^2^ and 9.2 × 10^4^ cfu/g (Additional file 2). In 2017, TVC of 1.3 × 10^4^ and 5.4 × 10^4^ cfu/g were found in fish cut and industrial fish, respectively (Additional file 5: Table S5).

In 2016, in all ingredients from feed producer C, TVC were between 2.6 × 10^3^ and 3.8 × 10^4^ cfu/g. In the same year, clostridial counts of 1.9 × 10^4^ cfu/g were found in fish cut and of 4 × 10^4^ cfu/g in poultry by-product (Additional file 3). In 2017, in blood samples all bacterial counts except clostridia were between 2.1 × 10^3^ and 4.4 × 10^5^ cfu/g (Additional file 6).

In both sampling years in all three feed producers, TVC of the ready-to-eat feed I varied between 9.8 × 10^4^ and 1.1 × 10^7^ cfu/g (Additional files 1, 2, 3, 4, 5 and 6). After storage overnight, TVC for the ready-to-eat feed II increased for all three producers to counts between 4.2 × 10^7^ and 1.4 × 10^9^ cfu/g.

In 2016, environmental samples from different surfaces, e.g. from the conveyor belts for the ingredients and for the final products, the mixer, the ready-to-eat feed silo, the distribution auger and the homogenizer showed TVC between 10^7^ and 10^9^ cfu/cm^2^ (Table 2) in all three feed producers. In 2017, lower TVC were detected from the environmental samples in all three feed producers (Table 2).

**Table 2:** Total viable counts of bacteria (cfu/cm^2^) from swab samples taken from the production facility at three feed producers (A–C) in 2016 and 2017

Feed producer (year)	Sampling location	Total viable bacteria (cfu/cm^2^)
A (2016)	Mixer 629	5.5 × 10^5^
	Auger 626	4.6 × 10^4^
	Conveyor belt 620	4.6 × 10^8^
	Conveyor belt 810	1.5 × 10^5^
	Final product silo	1.7 × 10^9^
A (2017)	Final product silo	1.5 × 10^3^
	Conveyor belt to homogenizer	4.5 × 10^2^
	Floor in room 1	2.7 × 10^6^
	Mincer	< 100
	Chain conveyer	< 100
B (2016)	Homogenizer	5.0 × 10^8^
	Conveyor belt to chopper	3.5 × 10^7^
	Mixer 1	1.5 × 10^9^
	Distribution auger	1.8 × 10^9^
	Conveyor belt to silo	1.2 × 10^5^
B (2017)	Final product silo 701	9.0 × 10^2^
	Final product silo 711	2.8 × 10^4^
	Conveyor belt 810	1.9 × 10^4^
	Conveyor belt 620	6.0 × 10^4^
	Distribution auger 626	5.0 × 10^2^
C (2016)	Auger 391	1.3 × 10^3^
	Auger 412	2.3 × 10^3^
	Auger 410	< 100
	Auger 436	4.0 × 10^7^
	Mixer 421	6.0 × 10^2^
C (2017)	Auger 410	2.9 × 10^3^
	Auger 391	1.0 × 10^2^
	Conveyor belt	1.8 × 10^5^
	Auger 412	2.0 × 10^2^
	Auger 422	7.2 × 10^2^

The TVC were not statistically significantly different between seasons (autumn 2016 versus spring 2017) (P > 0.05). However, there was significant difference between feed factories, i.e. factory A had significantly higher counts than factory B and C, whereas there was no difference between B and C.

In 2016, pork meat product from feed producer A and in 2017, both haemoglobin and pork liver from feed producer A and poultry mix from feed producer B were positive for MRSA. In 2017, *Salmonella* Typhimurium monophasic variant [4,5,12:i:-] was detected in a pork meat product from feed producer A. Neither MRSA nor *Salmonella* were detected in the ready-to-eat feed from any of the three feed producers. All samples of raw ingredients and ready-to-eat feed were influenza-negative.

### Fungal and mycotoxin analyses

Fungal analyses were made on 10 ready-to-eat feed samples from 2016. All samples contained Zygomycetes (*Absidia corymbifera, Mucor racemosus, Rhizopus oryzae, R. stolonifer* and/or *Syncephalastrum racemosum*), while three of the samples also contained *Aspergillus niger, A. flavus* and/or *A. glaucus*. In nine samples *Penicillium discolor* was detected whereas *P. polonicum* was detected in five and *P. albocoremium* in one sample (Table 3). The qualitative chemical analyses for mycotoxins showed a compound with the same molecular mass as aflatoxin B1 [(M+H)^+^ 313.0707 Dalton/Rt = 7.4 min] in all samples, but different retention time Rt = 7.9 min. One structure dihydro-demethyl-sterigmatocystin [(M+H)^+^ 313.0707 Dalton] a precursor to aflatoxins, fits the data. Furthermore, enniatins were found in six samples and one sample also contained deoxynivalenol (DON) (Additional file 7).

**Table 3:** Microbiological quality/fungal species present in samples of ready-to-eat feed from the three feed producers (A to C)

Producer	*Aspergillus* species	*Penicillium* species	*Absidia*, *Mucor*, *Rhizopus* and *Syncephalastrum* species
A	*A. niger, A. glaucus*	*P. discolor*	*Ab. corymbifera*
B	–	*P. discolor, P. polonicum*	*M. racemosus*
B	–	*P. albocoremium*	*M. racemosus*
B	–	*P. discolor, P. polonicum*	*M. racemosus, R. stolonifer*
B	–	*P. discolor*	*R. stolonifer*
B	–	*P. discolor, P. polonicum*	*M. racemosus, R. oryzae*
B	*A. flavus, A. glaucus*	*P. discolor, P. polonicum*	*S. racemosum*
B	*A. niger, A. glaucus*	*P. roqueforti*	*Ab. corymbifera, M. racemosus*
B	*A. flavus, A. niger*	*P. discolor, P. polonicum*	*S. racemosum*
C	*A. flavus, A. niger*	*P. discolor*	*Ab. corymbifera*

Fifty-six samples of raw ingredients and ready-to-eat feed were screened for the presence of mycotoxins and other bioactive fungal metabolites. The results (Table 4) showed large variation in occurrence as well as in concentrations of mycotoxins depending on the type of the raw materials. No fungal metabolites were detected in the ingredients of animal origin (e.g. blood meal or pig fat). Aflatoxins, citrinin, cyclopiazonic acid, patulin or ochratoxin were not detected in any of the ingredients of plant origin, however, high concentrations of dihydro-demethyl-sterigmatocystin (DHDMST, a precursor for aflatoxin) were detected in several ingredients of plant origin (e.g. sugar beet pulp and bio fibres). *Fusarium* metabolites were found in most of the ingredients of plant origin (Table 4). Fumonisins were detected in 7 out of the 31 plant-based ingredients, deoxynivalenol in 5 and zearalenone in six samples, whereas enniatins were detected in 12 of the 31 samples. Several ingredients and ready-to-eat feed samples contained more than one fungal metabolite or mycotoxin. No metabolites from Zygomycetes or *Penicillium* were found in the samples.

**Table 4:** Qualitative determination of mycotoxins and other biologically active metabolites present in samples of the different ingredients and ready-to-eat feed samples from the three feed producers (A to C)

Sample type (number of samples)	Aflatoxins	DHDMST^a^	Fumonisins	Enniatins	Other fungal mycotoxins/metabolites (number of samples)
Arbocel (n = 3)	0	1	1	1	0
Bio fiber (n = 1)	0	1	0	1	Zearalenone (1)
Corn gluten meal (n = 5)	0	1	3	0	Deoxynivalenol (2), Nivalenol (2), Zearalenone (3)
Extruded barley (n = 8)	0	1	2	4	Deoxynivalenol (1)
Extruded wheat (n = 4)	0	0	0	2	Deoxynivalenol (1), zearalenone (1)
Plant silage (n = 2)	0	0	1	0	Deoxynivalenol (1)
Soybean meal (n = 3)	0	0	0	2	Zearalenone (1)
Soybean oil (n = 4)	0	0	0	0	0
Sugar beet pulp (n = 5)	0	4	1	4	0
Blood meal (n = 2)	0	0	0	0	0
Haemoglobin (n = 1)	0	0	0	0	0
Meat product (n = 1)	0	0	0	0	0
Pigs fat (n = 2)	0	0	0	0	0
Ready-to-eat feed (n = 15)	0	3	0	2	0

^a^ Dihydro-demethyl-sterigmatocystin

## Discussion

The microbiological quality of feed is as important as the composition to ensure optimal growth and health of production animals. This study has shown that in both sampling years there is a great variety in bacterial counts between both similar and different types of raw ingredients and the three producers. Due to a continuous slaughter, by-products from slaughterhouses and the use of highly perishable raw ingredients like fish or poultry, these ingredients need preservation in order to maintain good microbiological quality when the feed is offered to the mink. Different treatments, like freezing (fish), heat treatment up to 90 °C (poultry) or acid treatment (silage of fish or spent laying hens) are used. However, high total viable counts (TVC) of bacteria up to 10^7^ cfu/g were found in both fish and poultry ingredients. Juokslahti [1] reported high bacterial counts in fish by-product and unpreserved slaughter blood in a study from 1979. Although both cooling and hygiene for by-products may have improved since then, we still found high or moderate high bacterial counts in fish products and in haemoglobin and other blood products from pigs. The high moisture and nutrient content of fish and poultry-based ingredients are favoring the growth of microorganisms [23]. Elevated temperature during transport and storage are likely the cause of the high bacterial counts of some ingredients seen in the present study. It is noteworthy that there were differences in viable counts between feed factories. It is not known whether this was due to differences in the quality of the raw materials the factories received or differences in procedures, such as treatment, transport or storage of raw materials and final products. Notwithstanding, the observation suggests that there is potential to improve the microbiological quality of the feed. This may be obtained by preventing growth of microorganisms or reducing their numbers e.g. using acidification or heat treatment, while others have suggested a beneficial effect of lactic acid fermentation [24].

To the authors’ knowledge, few studies have been published on the bacterial counts of freshly produced ready-to-eat mink feed products. When studying the effect of mink feed supplementation with sodium bentonite, Wlazlo et al. [25] reported TVC of 1.5–2.7 × 10^6^ cfu/g in ready-to-eat products. During this study, TVC between 10^4^ and 10^7^ cfu/g were found in the ready-to-eat feed. When we simulated time/temperature procedures (corresponding to normal feeding of farm mink) of the ready-to-eat feed by storing sampling containers with lid open at room temperature overnight, TVC increased up to 1.4 × 10^9^ cfu/g in ready-to-eat feed II. This result more or less corresponds to the bacterial concentration in a fully outgrown broth culture, and clearly shows the importance of maintaining the correct temperature of the ready-to-eat feed product during storage in the feed production facilities. Furthermore, bacteria can spread to the raw ingredients and ready-to-eat feed product during contact with contaminated feed handling equipment and storage containers. This condition applies not only to the feed producer, but also to mink farms, where cleaning and disinfection of feed silos and feeders should be included in daily routines. High bacterial counts of the environmental samples swapped from different surfaces at the feed production facilities with levels of 10^7^ to 10^9^ cfu/cm^2^ for TVC indicate low hygiene, poor cleaning and manufacturing practices. Thus, there is a risk of recontamination of raw ingredients and final ready-to-eat product by feed- and processing handling equipment and during storage. Several mink diseases caused by e.g. *Enterobacteriaceae*, *E. coli*, staphylococci and streptococci are caused by contaminated or spoiled feed [26].

Influenza A virus was not detected in the feed studied, but outbreaks of influenza in mink have previously been linked to feed as a potential source of infection [27]. Gagnon et al. [28] isolated H3N2 influenza A virus from Canadian mink and speculated uncooked pork meat by-products obtained from slaughterhouse facilities to be the source of transmission. As far as known, influenza viruses have never been detected directly in feed. However, it may be quite difficult to detect influenza viruses in feed as viruses do not multiply in the feed the way that bacteria do, and since only a very small proportion of each feed portion was tested.

In the present study, LA-MRSA was only found in raw ingredients: pork meat product, pork haemoglobin, pork lever and poultry mix. All samples of ready-to-eat feed were LA-MRSA negative. Hansen et al. [5] reported LA-MRSA in 20/108 (19%) of final mink feed products. LA-MRSA was also widely found on the paws and in the pharynx of the mink, and spill-over from pig production via raw slaughter by-product was strongly suggested to be the source [29]. In our case, all pork by-products were frozen and the poultry mix was heat treated when mixed into the final product. *Salmonella* Typhimurium was detected in a pork meat product, but not in the final products. Dietz et al. [2] concluded *Salmonella* Dublin-contaminated feed to be associated with an abortion storm in Danish mink and fox farms. *Salmonella* infections are zoonotic, which can lead to infections in both humans and animals. Our findings of pathogenic bacteria show that proper treatment and processing of raw ingredients are essential to reduce the risk of both the contamination of the ready-to-eat feed and a possible exposure to humans during handling the feed.

The results of the mycological analyses show that the ready-to-eat feed products were heavily contaminated with different fungal species, especially Zygomycetes, *Penicillium* and *Aspergillus* spp. Our results correspond well with a study on poultry feed, where *Penicillium* was the most frequent genus, followed by *Aspergillus* and *Mucor* [30].

Zygomycetes are fast growing, soil fungi [31] that produce up to 2 cm high hairy surface mycelium within days [32]. Food- and feed-borne Zygomycetes are not known to produce mycotoxins or other bioactive fungal metabolites [32, 33]. However, their presence is suggestive of a poor hygiene in the production facility. *Penicillium discolor* was the most dominant species in the ready-to-eat feed products followed by *P. polonicum*. They both tolerate low temperatures, low pH values and low water activities [21] and are commonly found on nuts and cereals in temperate climates [21, 32]. Their presence suggests contamination of stored, processed cereals (e.g. barley or wheat). *Aspergillus flavus* together with *A. niger* and *A. glaucus* were found in samples collected in August and November 2016. These fungi thrive at higher temperatures and lower water activities than the *Penicillium* species and are common on stored cereals, and on nuts, beans and cereals grown in the tropics or subtropics [21, 32]. *Aspergillus flavus* and *A. niger* are known mycotoxin producers [21], but only dihydro-demethyl-sterigmatocystin and *Fusarium* mycotoxins were detected in this study. Their presence suggests contamination in the field and/or during storage of cereals (e.g. maize). A number of *Fusarium* toxins were also detected in the barley, wheat and maize raw ingredients. These metabolites are usually produced in the field during growth and remain after the field fungi have been replaced with storage fungi like *Aspergillus* and *Penicillium*. Sugar beet pulp, together with the extruded cereals and corn gluten seem to be the raw ingredients that are most susceptible to fungal contamination and thereby contribute the most to the overall mycotoxin content in the ready-to-eat feed.

The results of the chemical analyses show that 12 out of 25 ready-to-eat feed samples contained dihydro-demethyl-sterigmatocystin, a precursor to aflatoxin [34], possibly originating from contaminated maize gluten in the feed [35]. Eight of the ready-to-eat feed samples also contained enniatins. These metabolites together with deoxynivalenol are produced by *Fusarium* species in cereals [21, 36] and may originate from either corn gluten or the extruded barley or wheat ingredients. The fungal metabolites detected in the ready-to-eat feed products may not in themselves be able to cause negative health effects in mink, but the additive or synergistic effects of fungal metabolites together with high amounts of bacteria in the feed may explain syndromes like wasting mink disease. There are some studies on mink and mycotoxins (e.g. aflatoxin and ochratoxin), but no studies on other biologically active metabolites (e.g. enniatins). On the other hand, there are several examples of pets that have become seriously ill after eating mycotoxin-containing feed products. Canadian dogs, which ate moldy foods containing penitrems A and roquefortine have died [37] while other dogs vomited, and developed diarrhea and liver failure after having eaten molded cereal products [38]. Cats fed with T2 toxin, commonly found in moldy corn, contracted leukopenia (decreasing white blood cells), bloody stools and ataxia of the hind body [39]. However, the above studies did not take any “cocktail effects” of either bacteria/mycotoxin or mycotoxin/mycotoxin into consideration in the complexity of the problem.

Mycotoxins make up only a small part of the many biologically active metabolites molds can secrete into mink feed. Different metabolites have different biological activity depending on dose: few are acute toxic, some have an estrogen-like effect, others cause liver and kidney damage and others again are carcinogenic. A group of fungal metabolites are also able to suppress the immune system [40], which leads to a higher risk of bacterial and viral infections, which in turn results in increased miscarriage and higher antibiotics consumption. It is therefore necessary to prevent and control both bacterial and mold contamination in both raw materials and final ready-to-eat feed products.

## Conclusions

Frequent microbiological control is important as many raw ingredients of mink feed have a very high number of bacteria and fungi and thus a short shelf life. The analytical methods have a natural limitation, as the sample volume is very small compared to the total production volume and microbial or chemical hotspots can easily be missed. As the raw material composition is constantly changing, potential cocktail effects should be considered when introducing new types of raw materials. Bacteria and fungi can spread to feed through contact with contaminated feed-handling equipment and grow in storage containers. Proper cleaning of equipment and proper timing of handling, storage, processing of ingredients and of the final products are essential to guarantee a high quality feed and to prevent or control infections in mink as well as the risk of exposure to humans from handling of mink feed. This study has shown that ready-to-eat mink feed can contain a cocktail of mycotoxins and bacteria, which may not in themselves cause clinical disease in the animals, but which may affect the overall health and well-being of the mink. These synergies may play an important role for the appearance of new disease complexes with unknown etiology.

## Supplementary information

**Additional file 1.** Microbiological quality/bacterial counts in raw ingredients of animal origin and ready-to-eat feed at producer A in 2016.

**Additional file 2.** Microbiological quality/bacterial counts in raw ingredients of animal origin and ready-to-eat feed at producer B in 2016.

**Additional file 3.** Microbiological quality/bacterial counts in raw ingredients of animal origin and ready-to-eat feed at producer C in 2016.

**Additional file 4.** Microbiological quality/bacterial counts in raw ingredients of animal origin and ready-to-eat feed at producer A in 2017.

**Additional file 5.** Microbiological quality/bacterial counts in raw ingredients of animal origin and ready-to-eat feed at producer B in 2017.

**Additional file 6.** Microbiological quality/bacterial counts in raw ingredients of animal origin and ready-to-eat feed at feed producer C in 2017.

**Additional file 7.** Qualitative determination of mycotoxins and other biologically active metabolites present in samples of ready-to-eat feed from the three feed producers (A to C).

## Data Availability

The datasets during and/or analysed during the current study are available from the corresponding author on reasonable request.
