# Supplementary material for: Microbiological quality of mink feed raw materials and feed production area
Source: Acta Vet Scand. 2019 Nov 21;61:56. doi: 10.1186/s13028-019-0489-6 (PMC6873557; doi:10.1186/s13028-019-0489-6)
Supplement: Supplementary file 1 — Additional file 1. Microbiological quality/bacterial counts in raw ingredients of animal origin and ready-to-eat feed at producer A in 2016. [file 13028_2019_489_MOESM1_ESM.docx]

**Additional file 1.** Microbiological quality/bacterial counts in raw ingredients of animal origin and ready-to-eat feed at producer A in 2016

| Sample type | Treatment of samples | Total viable counts (cfu/g) | *Enterobacteriaceae* (cfu/g) | Clostridia (cfu/g) | *E. coli* (cfu/g) | Staphylococci (cfu/g) | pH |
| --- | --- | --- | --- | --- | --- | --- | --- |
| Poultry by-product, Sweden | heat treated  80-90 ºC | 2.2 × 10^5^ | 3.6 × 10^3^ | <100 | 2 × 10^3^ | 2.0 × 10^5^ | 6.5 |
| Poultry by-product, Germany | heat treated  80-90 ºC | 1.8 × 10^4^ | 4.7 × 10^3^ | 1.0 × 10^2^ | 3 × 10^2^ | 1.0 × 10^3^ | 4.5 |
| Fish cut 7-11% fat | fresh/frozen | >10^9^ | >10^9^ | <100 | 1.7 × 10^4^ | 9.0 × 10^2^ | 6.5 |
| Fish cut | fresh/frozen | 3.6 × 10^6^ | 1.7 × 10^4^ | <100 | 2.7 × 10^3^ | 4.5 × 10^4^ | 6.6 |
| Pork meat product | fresh/frozen | >10^9^ | >10^9^ | <100 | 9.2 × 10^3^ | 1.5 × 10^4^ | 7.2 |
| Pork slaughter-mix | heat treated  75-90 ºC | >10^9^ | >10^9^ | 4.0 × 10^5^ | 9.4 × 10^3^ | 1.0 × 10^3^ | 7.4 |
| Pork haemoglobin | fresh/frozen | >10^9^ | >10^9^ | <100 | 4.0 × 10^2^ | 1.8 × 10^3^ | 7.2 |
| Ready-to-eat feed I |  | >10^9^ | >10^9^ | 3.0 × 10^3^ | 6.7 × 10^3^ | 1.9 × 10^3^ | 5.7 |
| Ready-to-eat feed II |  | >10^9^ | 2.6 × 10^4^ | 7.0 × 10^3^ | 1.4 × 10^4^ | 3.4 × 10^5^ | 4.8 |
