# Supplementary material for: Microbiological quality of mink feed raw materials and feed production area
Source: Acta Vet Scand. 2019 Nov 21;61:56. doi: 10.1186/s13028-019-0489-6 (PMC6873557; doi:10.1186/s13028-019-0489-6)
Supplement: Supplementary file 2 — Additional file 2. Microbiological quality/bacterial counts in raw ingredients of animal origin and ready-to-eat feed at producer B in 2016. [file 13028_2019_489_MOESM2_ESM.docx]

**Additional file 2.** Microbiological quality/bacterial counts in raw ingredients of animal origin and ready-to-eat feed at producer B in 2016

| Sample type | Treatment of  samples | Total viable  counts (cfu/g) | *Enterobacteriaceae*  (cfu/g) | Clostridia  (cfu/g) | *E. coli* (cfu/g) | Staphylococci  (cfu/g) | pH |
| --- | --- | --- | --- | --- | --- | --- | --- |
| Poultry by-product and mix | heat treated  80-90 ºC | 7.2 × 10^2^ | <100 | <100 | <100 | <100 | 6.5 |
| Spent hens | acid treated | 7.1 × 10^3^ | <100 | 5.0 × 10^3^ | 100 | <100 | 2.9 |
| Industrial fish 8-12% fat | fresh/frozen | 5.2 × 10^4^ | <100 | 1.8 × 10^2^ | <100 | 4.0 × 10^3^ | 6.5 |
| Industrial fish 5-8% fat | fresh/frozen | 1.1 × 10^3^ | <100 | <100 | <100 | <100 | 7.8 |
| Fish cut | fresh/frozen | 9.2 × 10^4^ | <100 | 9.0 × 10^2^ | 100 | <100 | 7.2 |
| Pork slaughter-mix | heat treated  75-90 ºC | 7.9 × 10^2^ | <100 | <100 | <100 | <100 | 6.2 |
| Pork blood meal | dry | 3.5 × 10^4^ | <100 | <100 | <100 | 8.0 × 10^3^ | 7.2 |
| Ready-to-eat feed I |  | 9.8 × 10^4^ | 1.3 × 10^2^ | 1.7 × 10^2^ | 3.0 × 10^2^ | 8.0 × 10^3^ | 5.6 |
| Ready-to-eat feed II |  | 5.5 × 10^7^ | 4.1 × 10^3^ | <100 | 1.4 × 10^3^ | 1.9 × 10^3^ | 5.6 |
