# Supplementary material for: Microbiological quality of mink feed raw materials and feed production area
Source: Acta Vet Scand. 2019 Nov 21;61:56. doi: 10.1186/s13028-019-0489-6 (PMC6873557; doi:10.1186/s13028-019-0489-6)
Supplement: Supplementary file 3 — Additional file 3. Microbiological quality/bacterial counts in raw ingredients of animal origin and ready-to-eat feed at producer C in 2016. [file 13028_2019_489_MOESM3_ESM.docx]

**Additional file 3.** Microbiological quality/bacterial counts in raw ingredients of animal origin and ready-to-eat feed at producer C in 2016

| Sample type | Treatment of samples | Total viable counts (cfu/g) | *Enterobacteriaceae*  (cfu/g) | Clostridia  (cfu/g) | *E. coli* (cfu/g) | Staphylococci  (cfu/g) | pH |
| --- | --- | --- | --- | --- | --- | --- | --- |
| Industrial fish | fresh/frozen | 2.6 × 10^3^ | <100 | <100 | <100 | <100 | 5.9 |
| Fish cut | fresh/frozen | 3.8 × 10^4^ | <100 | 1.9 × 10^4^ | <100 | <100 | 7.1 |
| Fish | fresh/frozen | 9.8 × 10^3^ | <100 | <100 | <100 | 1.0 × 10^3^ | 6.8 |
| Poultry by-product and mix, Denmark | heat treated  80-90 ºC | 3.4 × 10^3^ | <100 | 6.0 × 10^3^ | <100 | <100 | 6.3 |
| Poultry by-product and mix, Germany | heat treated  80-90 ºC | 3.5 × 10^4^ | 10^2^ | 1.4 × 10^3^ | <100 | 4.0 × 10^4^ | 6.3 |
| Pork slaughter- mix | heat treated  75-90 ºC | 7.8 × 10^3^ | ND^1^ | ND^1^ | ND^1^ | ND^1^ | 4.6 |
| Fat meal | dry | <100 | ND^1^ | ND^1^ | ND^1^ | ND^1^ | 5.9 |
| Meat meal | dry | 2.9 × 10^4^ | ND^1^ | ND^1^ | ND^1^ | ND^1^ | 5.7 |
| Blood meal | dry | 2.8 × 10^4^ | ND^1^ | ND^1^ | ND^1^ | ND^1^ | 7.1 |
| Pork fat | heat treated  70 ºC | <100 | ND^1^ | ND^1^ | ND^1^ | ND^1^ | ND^1^ |
| Ready-to-eat feed I |  | 1.3 × 10^6^ | 9.4 × 10^2^ | 9.0 × 10^2^ | 7.0 × 10^2^ | <100 | 5.7 |
| Ready-to-eat feed II |  | 4.2 × 10^7^ | <100 | <100 | 1.0 × 10^2^ | 1.0 × 10^3^ | 5.0 |

^1^ND: not determined
