# Supplementary material for: Microbiological quality of mink feed raw materials and feed production area
Source: Acta Vet Scand. 2019 Nov 21;61:56. doi: 10.1186/s13028-019-0489-6 (PMC6873557; doi:10.1186/s13028-019-0489-6)
Supplement: Supplementary file 5 — Additional file 5. Microbiological quality/bacterial counts in raw ingredients of animal origin and ready-to-eat feed at producer B in 2017. [file 13028_2019_489_MOESM5_ESM.docx]

**Additional file 5.** Microbiological quality/bacterial counts in raw ingredients of animal origin and ready-to-eat feed at producer B in 2017

| Sample type | Treatment of  samples | Total viable  counts (cfu/g) | *Enterobacteriaceae*  (cfu/g) | Clostridia  (cfu/g) | *E. coli* (cfu/g) | Staphylococci  (cfu/g) | pH |
| --- | --- | --- | --- | --- | --- | --- | --- |
| Industrial fish | fresh/frozen | 5.4 × 10^4^ | <100 | <100 | <100 | <100 | 6.9 |
| Fish cut | fresh/frozen | 1.3 × 10^4^ | <100 | <100 | <100 | <100 | 7.1 |
| Fish silage | acid treated | <100 | <100 | 2.0 × 10^2^ | <100 | <100 | 3.0 |
| Pork slaughter-mix | heat treated  75-90 ºC | <100 | <100 | <100 | <100 | <100 | 6.6 |
| Poultry by-product  and mix | heat treated  80-90 ºC | <100 | <100 | <100 | <100 | <100 | 6.4 |
| Poultry product, processed, low fat | heat treated  80-90 ºC | <100 | <100 | <100 | <100 | 1.0 × 10^3^ | 6.3 |
| Ready-to-eat feed I |  | 8.0 × 10^4^ | 2.3 × 10^3^ | 2.7 × 10^2^ | 3.3 × 10^4^ | 1.0 × 10^3^ | 3.4 |
| Ready-to-eat feed II |  | 2.7 × 10^8^ | 7.9 × 10^6^ | 1.3 × 10^2^ | 5.4 × 10^5^ | 1.5 × 10^5^ | 5.9 |
