# Supplementary material for: Microbiological quality of mink feed raw materials and feed production area
Source: Acta Vet Scand. 2019 Nov 21;61:56. doi: 10.1186/s13028-019-0489-6 (PMC6873557; doi:10.1186/s13028-019-0489-6)
Supplement: Supplementary file 7 — Additional file 7. Qualitative determination of mycotoxins and other biologically active metabolites present in samples of ready-to-eat feed from the three feed producers (A to C). [file 13028_2019_489_MOESM7_ESM.docx]

**Additional file 7.** Qualitative determination of mycotoxins and other biologically active metabolites present in samples of ready-to-eat feed from the three feed producers (A to C)

| Producer | Aflatoxins | DHDMST^1^ | Fumonisins | Enniatins | Other fungal mycotoxins/metabolites |
| --- | --- | --- | --- | --- | --- |
| A | 0 | 0 | 0 | 0 | 0 |
| B | 0 | 1 | 0 | 1 | 0 |
| B | 0 | 1 | 0 | 0 | 0 |
| B | 0 | 1 | 0 | 1 | 0 |
| B | 0 | 1 | 0 | 1 | 0 |
| B | 0 | 1 | 0 | 0 | 0 |
| B | 0 | 1 | 0 | 1 | 0 |
| B | 0 | 1 | 0 | 1 | 0 |
| B | 0 | 1 | 0 | 1 | Deoxynivalenol |
| C | 0 | 1 | 0 | 1 | 0 |

^1^ dihydro-demethyl-sterigmatocystin
